# Menthol as a Local Anæsthetic in Nose and Throat

**Published:** 1886-11

**Authors:** 


Menthol as a Local Anæsthetic in Nose and Throat.—Dr. Rosenberg finds that a fifty per cent. alcoholic solution produces anæsthesia in those parts, lasting from twenty to thirty minutes. It is not so complete as cocaine, but is a valuable supplement to the latter drug.—*Berlin Med. Wochenschrift.*

Dr. E. Baisrtrocchi has demonstrated the existence of a lympathic gland of the heart. The gland is situated where the fold at the beginning of the aorta touches the pulmonary artery. He finds the gland to be constant in many of the lower animals and has observed it five times in the human subject.

---

Professor Pecholier of Montpellier recommends hot baths and quinine for the abortive treatment of typhoid fever. A homœpathic practitioner accomplishes the same result by giving a drop of rhus tox. 3 x., or about 1-1000 of a drop of the tincture every two hours. Take your choice.

---

Argentine Republic has established a micro-biological institute to be placed under the supervision of the Medical Faculty of the State University. The object is to be provided with the means for the preventive inoculation for rabies and to make investigations in bacteriology.

---

Dr. W. Strudwick, of Hilsboro, N. C., recommends the administration of quinine in enormous doses, 100 grains every hour, for the cure of traumatic tinnitus. He has treated three cases in this manner successfully.

---

Dr. J. E. Emerson, of Detroit, cures neuralgia, of a rheumatic or malarial origin, with salicylate of cinchonidia in five grain doses, three or four times a day. In rheumatic neuralgia it acts almost as a specific.

---

Very wisely the church exhibits its conservative tendencies in the matter of cremation. The Pope has issued a Bull prohibiting Roman Catholics from joining societies advocating cremation.

---

Dr. Nivison of Burdett, N. Y., reports a case in which the first three fingers, completely severed by an axe, were reunited and the functions perfectly restored.

---

The New York Academy of Medicine has received a legacy of $25,000 from the late Mr. Woerishoffer. Prof. A. Jacobi will formally present the gift.

A MOVEMENT is on foot in Paris to place all the hospitals and asylums now controlled by religious bodies in the hands of the municipal government.

---

FOR the relief of migraine Dr. Brunton recommends salicylate of soda in two to three-grain doses every quarter or half hour until the pain disappears.

---

A PENSION of $1,200 per annum has been granted the widow of Dr. Von Gudden, who lost his life while caring for the insane king of Bavaria.

---

CONTRIBUTIONS are desired for the relief of the Medical College of South Carolina, whose building was destroyed by the recent earthquake.

---

TYPHOID FEVER is now more prevalent in Buffalo than it has been for many years or at least since 1881.

---

A DONATION of $200,000 has been given to found a homœopathic hospital in Detroit, Mich.

